# The Decoupling of Hardness and Elastic Modulus in Ti-Based Metallic Glasses Induced by Elastic Pretreatment

**DOI:** 10.3390/ma19102024

**Published:** 2026-05-13

**Authors:** Anwei Wang, Yang Wang, Lei Hou, Hanxiao Sun, Xinyi Xie, Jingbo Duan, Chen Li, Yansen Li

**Affiliations:** 1 School of Astronautics, Harbin Institute of Technology, Harbin 150001, Chinahoulei@hit.edu.cn (L.H.); 2 SANY Energy Equipment Co., Ltd., Zhuzhou 412005, China; 3 SANY Petroleum Intelligent Equipment Co., Ltd., Beijing 102200, China; 4 Department of Basic Teaching, Shijiazhuang Institute of Railway Technology, Shijiazhuang 050041, China; 5 Department of Engineering Mechanics, Shijiazhuang Tiedao University, Shijiazhuang 050043, China; 6 Hebei Key Laboratory of Mechanics of Intelligent Materials and Structures, Shijiazhuang Tiedao University, Shijiazhuang 050043, China

**Keywords:** nanoindentation, hardness, elastic modulus, shear transformation zone, metallic glass

## Abstract

In this paper, the elastic precompression method is employed as a pretreatment technique to investigate the evolution and characteristics of the micro-mechanical properties of metallic glasses. Nanoindentation analysis indicates that pre-compression treatment leads to structural rearrangement within the material, which in turn influences the nucleation and propagation of shear bands, resulting in a transition of serrated flow from a step-like to a wave-like pattern under a 400 MPa load held for 75 min. Crucially, precompression triggers a unique “decoupling” response: hardening alongside elastic softening. Further, this structural evolution is evidenced by the shear transition zone volume calculated using the jump rate method. The shear transition zone volume exhibits a nonlinear trend, initially increasing and then decreasing with increasing compressive strength and holding time, which reflects the kinetic competition mechanism between local shear instability and coordinated atomic rearrangement that arises under precompression. This study elucidates the effect of elastic precompression treatment on the micromechanical properties of a Ti-based metallic glasses, providing a reference for the optimization of plasticity in metallic glasses.

## 1. Introduction

Metallic glasses (MGs), with their unique topological structure characterized by long-range disorder and short-range order, exhibit yield strengths, elastic limits, and exceptional corrosion resistance that surpass those of conventional crystalline metals under near-equilibrium conditions [1,2], demonstrating immense potential for engineering applications in the aerospace and Micro-Electro-Mechanical Systems (MEMS) fields. However, at room temperature, the plastic flow behavior of amorphous solids exhibits extreme non-uniformity. Deformation loads are often highly localized within extremely narrow shear bands. This severe shear localization induces significant strain softening in the material, leading to an inherent bottleneck characterized by a lack of macroscopic plasticity and catastrophic brittle fracture [3,4]. Achieving the effective, controlled initiation and dispersed distribution of shear bands has become a scientific challenge that urgently needs to be addressed in the field of amorphous mechanics, as well as a key prerequisite for advancing its engineering applications.

To modulate plastic flows, researchers have proposed various strategies, including composition optimization [5,6,7,8], second-phase embedding [9,10], and energy state regulation [11,12,13]. Composition design has been shown to alter glass-forming ability, elastic modulus, and shear-band stability by tuning atomic-size mismatch, mixing enthalpy, and configurational complexity [5,6,7,8]. Zuo et al. achieved efficient optimization of the composition of MGs using a co-evolution algorithm, with correlation coefficients of 0.927 and 0.924 for the prediction of glass transition temperature and crystallization temperature. Mahjoub et al. [8] added small amounts of Cu and Ag to Pd-Si MG, significantly improving its stability and compressive plasticity, which they attributed to a reduction in the energy of the electronic states. Second-phase or heterogeneous-structure strategies can introduce additional interfaces and stress concentrators, thereby promoting multiple shear-band nucleation and suppressing catastrophic localization [9,10]. Li et al. [9] reported that a Ni_26_Mo alloy with a nanocrystalline amorphous composite structure (comprising an amorphous/nanocrystalline transition zone and a network of nanocrystalline interfaces) exhibits higher hardness than fully nanocrystalline alloys. Energy state regulation, such as annealing, cryogenic treatment, or mechanical loading, can further adjust the free volume distribution and relaxation state of MGs [11,12,13]. Shin et al. [12] found that sub-T_g_ annealing of the Cu_50_Zr_50_ MG below its glass transition temperature significantly improves its corrosion resistance in chloride ion media; this is attributed to the relaxation of thermally activated short-range ordered structures and the formation of energetically stable icosahedral clusters. Among these, the “mechanical rejuvenation” effect, achieved through the application of external stress fields, is regarded as a highly promising method for microstructural control. This effect induces the topological rearrangement of free volume and a fine-tuned distribution of residual stress fields within the material through precompression or cyclic loading [14,15,16]. Wang et al. [16] studied the Zr_41.2_Ti_13.8_Cu_12.5_Ni_10_Be_22.5_ MG and found that reducing the cooling rate at the glass transition temperature (T_g_) from 64 K·s^−1^ to 45 K·s^−1^ increased the free volume and the number of icosahedral clusters, thereby gradually increasing the plastic strain during compression testing without a loss of yield strength. While pre-compression has been shown to delay fracture in Zr- and Cu-based systems by promoting multiple shear band nucleation, the literature reveals complex kinetic behaviors and sometimes contradictory conclusions, the hardening/embrittlement and plasticity-enhancing effects induced by pretreatment exhibit distinct decoupling characteristics across different systems [3]. This discrepancy essentially reflects the nonlinear response of prestressing parameters to the modulation of the potential energy landscape. More importantly, while current research has largely focused on the macroscopic phenomenological mechanical characteristics, there remains a lack of systematic and in-depth physical understanding regarding how precompression reshapes deformation mechanisms at the nanoscale and even the atomic scale, particularly concerning the quantitative control mechanisms governing the activation energy barriers of shear transition zones (STZs) and the discrete dynamics of serrated flow [4,17], which severely limits the control of amorphous plasticity instability behavior.

Plasticity in MGs fundamentally arises from coordinated atomic rearrangements within STZs, which evolve into macroscopic shear bands. At the nanoscale, nanoindentation serves as a precise probe for detecting localized mechanical responses, capable of capturing discrete displacement jumps (Pop-in) in the load–displacement curve, which directly correspond to the transient physical events of shear band nucleation and expansion [18]. A quantitative analysis of serrated flow characteristics (such as serration frequency and stress drop amplitude) provides key criteria for evaluating the resistance to shear band activation and the evolution of slip. Furthermore, by combining the Johnson–Samwer cooperative shear model with variable-rate indentation experiments to quantitatively determine the STZ volume [19], this study provides a theoretical basis for quantifying the influence of structural evolution on the cooperativity of plasticity units [20].

This study focuses on Ti-based MGs and, through systematic nanoindentation experiments, comprehensively investigates the multiscale regulatory effects of precompression strength and dwell time on microstructural mechanical evolution. The study focuses on the evolution of serrated flow dynamics, the STZ characteristic volume, and activation energy barriers, aiming to reveal the intrinsic physical mechanisms underlying the evolution of structural inhomogeneities induced by mechanical pretreatment, by correlating microscopic mechanical parameters with structural energy states. This work provides a scientific basis and technical reference for optimizing the plasticity of MGs through mechanical pretreatment.

## 2. Materials and Methods

The mixture was remelted at least five times to ensure material homogeneity, resulting in the preparation of a Ti_40_Zr_25_Cu_9_Ni_8_Be_18_ master alloy. The master alloy was then cast into rod samples with a diameter of 5 mm and a height of 150 mm using the copper mold upper suction casting method.

We cut the test specimens into 5 mm high cylinders and performed preliminary grinding to ensure that the specimen surfaces are free of visible scratches. The precompression tests were conducted on a CMT5105 floor-standing dual-column small-door microcomputer-controlled electronic universal testing machine (SHENZHEN SANSI TESTING INSTRUMENT Co., Ltd., Ningbo City, China). The tests were performed in displacement control mode, and the force measurement error of the equipment complied with the requirements of the ASTM E9-19 standard [21] for room-temperature compression testing of metallic materials, with a measurement error of ≤±1%. The target precompression strengths were set to 0, 400, and 800 MPa, with a dwell time of 75 min for each. A control group was added with a dwell time of 150 min at 400 MPa. The corresponding specimens were designated as as-cast, 400T1, 400T2, and 800T1; the test has been repeatedly validated, and the results are accurate and reliable. After precompression treatment, the specimens were left to stand for 12 h to ensure sufficient atomic diffusion between the shear bands.

**Figure 1:**
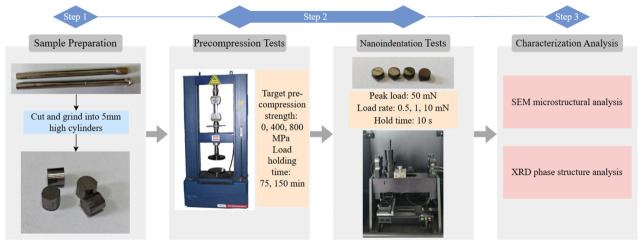
Experimental Flowchart.

Prior to the nanoindentation test, the samples were ground and polished to achieve a smooth, flat surface, and then cleaned with anhydrous ethanol to thoroughly remove any residual contaminants. The nanoindentation test was performed using a KLA iMicro indenter equipped with a Berkovich indenter (KLA Corporation, Milpitas, CA, USA), the tests were conducted in strict accordance with the international standards ISO 14577-1:2015 [22] and ASTM E2546-2023 [23] for instrumented indentation testing. The peak load was set to 50 mN, with loading rates of 0.5, 1 and 10 mN/s. The hold time was set to 10 s for each loading rate to eliminate the effects of thermal drift. The unloading rate matched the loading rate. Each set of data was repeated five times to ensure the reliability of the experimental results. The indentation morphology of the samples was characterized by scanning electron microscopy (SEM, Zeiss Gemini SEM 300, Carl Zeiss Co., Ltd., Oberkochen, Germany). The structure of the samples before and after treatment was verified by X-ray diffraction (XRD) using Cu Kα radiation. The experimental flowchart is shown in Figure 1:

The load–depth curves in this study were acquired directly from the experimental setup; nanoindentation hardness and elastic modulus were calculated using the classical Oliver–Pharr method [24]; the serrated flow characteristics of the load–displacement curves and the characteristic volume and activation energy barrier of the STZ, were quantitatively calculated and inverted based on the Johnson–Samwer Cooperative Shear Model (CSM) [25].

## 3. Results and Discussion

**Figure 2:**
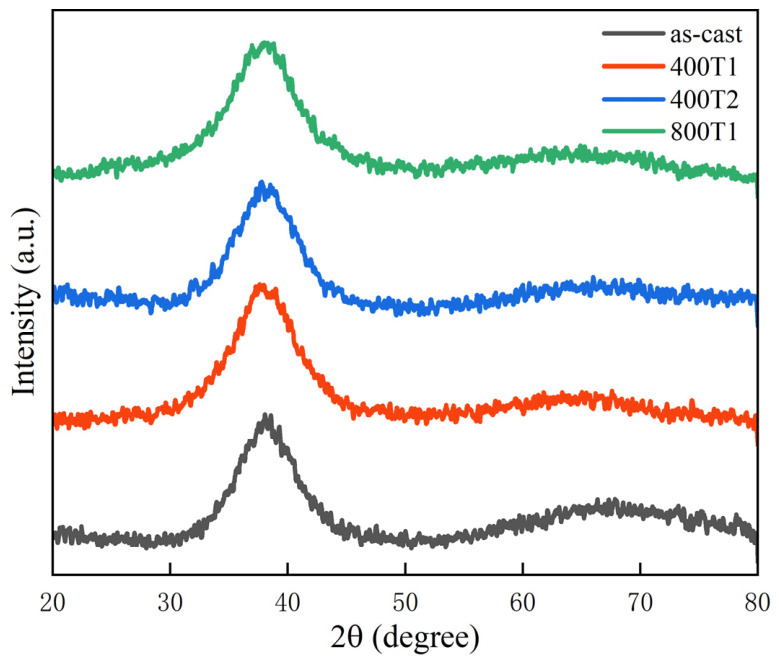
XRD patterns of the as-cast and elastically pre-compressed specimens.

Figure 2 presents X-ray diffraction (XRD) patterns for as-cast and precompression specimens. All samples exhibit broad diffraction halos with no sharp crystalline peaks, confirming their fully amorphous structure, indicating that the elastic pretreatment did not induce crystallization.

**Figure 3:**
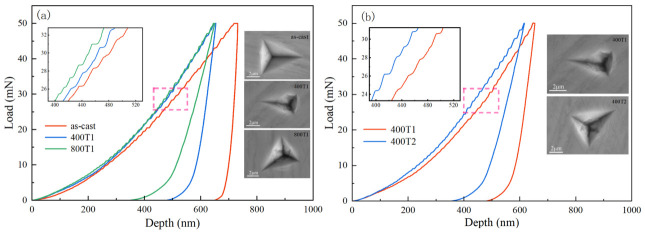
(**a**) Load–depth curves for different precompression strengths; (**b**) Load–depth curves for different precompression durations.

Figure 3 shows representative load–depth (P-h) curves at a loading rate of 0.5 mN/s; the inset shows a magnified view of the curve and the indentation micromorphology. All curves exhibit characteristic intermittent displacement jumps during the loading phase, known as “serrated flow” or “pop-in” events [20]. This discontinuous plastic response essentially reflects the localized, instantaneous restructuring of the internal structure of the Ti-based amorphous matrix and the abrupt release of strain energy; it serves as a key criterion for characterizing the microstructural inhomogeneities and the evolution kinetics of shear bands.

To highlight serration features, the curves in the magnified image were horizontally shifted. The results show that the as-cast specimen (0 MPa) exhibits highly discrete, “step-like” serrations with pronounced step features, suggesting isolated slip in the main shear band; in sharp contrast, the 400 MPa pretreatment smooths the curve, transforming it into a “wavy” rheological behavior characterized by high frequency and low amplitude, suggesting that the nucleation of multiple shear bands induced by precompression effectively refined the plastic deformation units. However, when the stress was further increased to 800 MPa, the dispersion of the serrations rebounded, falling between the two previously mentioned values. Figure 3b further illustrates the effect of the time dimension: as the dwell time increased from 75 min to 150 min, both the maximum indentation depth (*h_max_*) and the residual depth decreased monotonically, and the serration features coarsened from fine, wavy patterns back to coarse, stepped patterns.

Furthermore, as shown in Figure 3a, the *h_max_* value for the as-cast specimen is 731.93 nm. The continuous decrease in *h_max_* as the precompression load is incrementally increased confirms the steady improvement in the material’s resistance to penetration. It is worth noting that when the precompression strength was increased from 400 MPa to 800 MPa, the reduction in *h_max_* significantly narrowed. This phenomenon indicates that the surface-strengthening effect induced by precompression does not increase linearly but gradually approaches physical saturation as the stress level rises, with the hardening rate exhibiting a pronounced diminishing returns effect.

**Figure 4:**
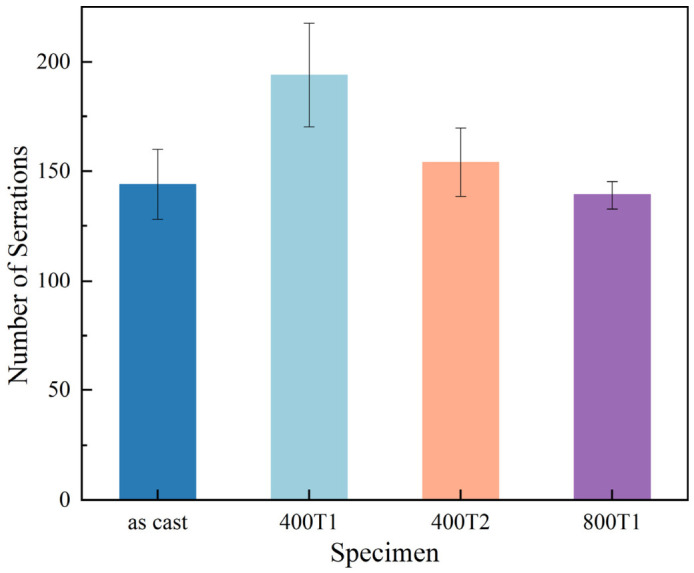
Statistics on the number of serrations under different conditions.

Figure 4 presents a quantitative analysis of the frequency of serration events. Experimental measurements show that the total number of serrations for the as-cast, 400T1, 400T2, and 800T1 samples were 144, 194, 154, and 139, respectively. The frequency of serrations in the 400T1 sample exhibits a distinct peak characteristic; in contrast, the number of serrations in the as-cast, 400T2, and 800T1 groups shows a converging trend, with minimal differences among them. This evolutionary pattern clearly reflects the sensitivity of serrated flow dynamics to the magnitude of the precompression stress and the dwell time. Based on the physical logic that the frequency of serrated flow is positively correlated with the density of shear band nucleation around the indentation field [26], and in conjunction with the theoretical framework that the plastic rheology of MGs is governed by the number and spatial distribution of shear bands, it can be inferred that the 400T1 process induces the highest density of localized shear carriers within the material. This proliferation and dispersion of shear bands not only effectively reduces the degree of localized plastic deformation but also makes the intermittent response characteristics during the micro-indentation process most pronounced, thereby conferring superior plasticity control potential on the material at the macroscopic level.

**Figure 5:**
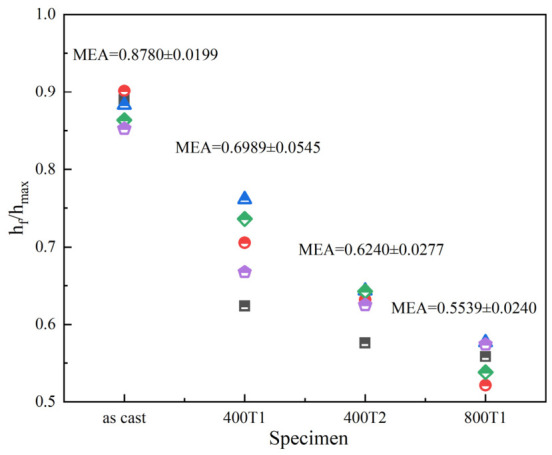
The value of *h_f_*/*h_max_* of different specimens (Each symbol in the figure represents a single measurement from five independent nanoindentation tests conducted under the same conditions; the MEA value indicated is the mean ± standard deviation of the five measurements for the corresponding specimen).

To further probe the microstructural deformation of Ti-based MGs, this study introduced the ratio of residual indentation depth to maximum indentation depth (*h_f_*/*h_max_*) as an evaluation metric to quantify the material’s elastic recovery capacity and plastic contribution. The results are shown in Figure 5.

The experimental data shows that the *h_f_*/*h_max_* value for the as-cast specimen is 0.878 ± 0.0199, indicating that its loading response is dominated by plastic flow. As the precompression stress increases, this ratio decreases significantly, dropping to 0.5539 ± 0.0240 under the 800T1 condition. Although the samples still exhibit plastic deformation characteristics macroscopically, the proportion of elastic recovery has increased significantly, showing a trend toward quasi-elastic behavior. Further comparison of the 400T1 and 400T2 samples revealed that, as the dwell time was extended from 75 min to 150 min, the *h_f_*/*h_max_* value decreased further from 0.6989 ± 0.0545 to 0.6240 ± 0.0277. The aforementioned evolutionary pattern clearly indicates that elastic precompression treatment effectively alters the mechanical response and deformation mechanism of MGs under localized stress fields by finely tuning the microstructure of the amorphous matrix.

The hardness of MGs is a key mechanical parameter that measures their ability to resist localized plastic deformation, and it is closely related to the packing density and defect distribution of their microscopic atomic structure [27]. Young’s modulus essentially reflects the bonding strength between atoms and their resistance to elastic strain. A comprehensive analysis of the evolution patterns of hardness and elastic modulus can provide deeper insights into the microstructural evolution induced by elastic precompression treatment.

**Figure 6:**
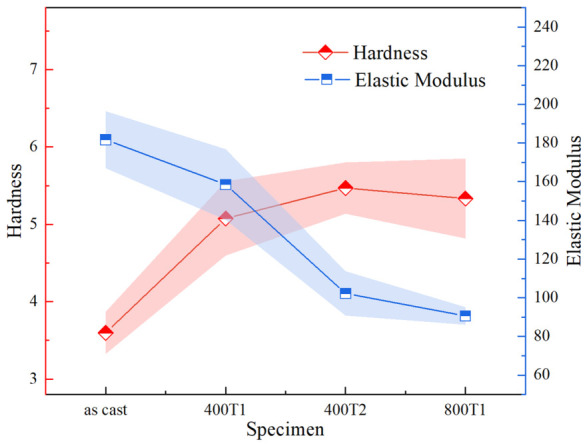
The hardness and elastic modulus curves of different specimens.

As shown in Figure 6, the hardness of the as-cast sample is at its lowest level; as the magnitude of the precompression stress and the dwell time increase, the sample hardness exhibits a clear upward trend. In contrast, the elastic modulus gradually decreases as the precompression conditions are intensified. This divergent trend of “increasing hardness and decreasing modulus” reveals the complex nature of the metastable structural reorganization induced in the amorphous matrix by elastic precompression:

The decrease in elastic modulus validates the structural rejuvenation mechanism: the mechanical pumping of work from an external stress field drives the material to transition to a metastable state at a higher energy level within the potential energy landscape. This process induces localized disorder in atomic packing density and slight changes in the average coordination number, resulting in a decrease in the slope of the interatomic potential energy curve. Macroscopically, this manifests as a weakening of the average atomic bonding force and a softening of the Young’s modulus.

The anomalous increase in hardness can be attributed to local structural rearrangement and an increase in the nucleation energy barrier. Unlike simple structural relaxation, elastic precompression induces a spatially inhomogeneous redistribution of free volume, rather than merely a change in its overall content. The prestress field may induce localized densification in the “soft zones” of the original organization, which are highly susceptible to plastic instability, thereby eliminating some of the early instability sites; additionally, as shown in Table 1 below, the pretreatment significantly increases the volume (Ω) of the STZ. According to the cooperative shear model, a larger STZ implies that initiating plastic flow requires overcoming a broader range of atomic cooperative resistance, i.e., it significantly increases the activation barrier for shear band nucleation. During nanoindentation, this “nucleation hardening” effect requires a larger external load to trigger localized plastic flow, thereby manifesting as an increase in macroscopic hardness.

In summary, the coexistence of this “modulus softening” induced by structural disorder and “plastic hardening” induced by an increase in the nucleation energy barrier fully demonstrates that elastic precompression treatment effectively alters the deformation mechanism of Ti-based MGs by finely regulating the distribution and dynamics of microstructural defects, thereby laying the microstructural foundation for enhancing their plasticity.

**Figure 7:**
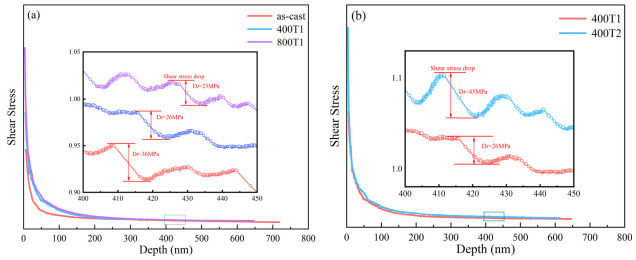
(**a**) The shear stress–depth curves for different precompression strengths; (**b**) the shear stress–depth curves for different precompression durations.

To further investigate the effect of precompression treatment on the serrated flow behavior of Ti-based MGs, the shear stress (*τ*)–depth (*h*) curves (Figure 7) were analyzed for different precompression treatments. Based on the Tabor relationship [28] and the von Mises yield criterion [29], the nanoindentation hardness H and the shear stress τ satisfy

(1) $$3\sqrt{3}\tau \approx H$$

According to the indentation depth measurement method proposed by Oliver and Pharr [24], the relationship between nanoindentation hardness *H* and indentation depth *h* is given by the following equation:

(2) $$H=\frac{P}{C\times h^{2}}$$

where *P* represents the load; C is a constant related to the indenter; and for the Berkovich indenter, the value is 24.56.

Combining Equations (1) and (2) yields the expression for shear stress:

(3) $$\tau =\frac{P}{24.56\times 3\sqrt{3}h^{2}}$$

As shown in Figure 7, the serrated flow behavior is evident not only in the load–depth curve but also in the shear stress–depth curve, which exhibits distinct serrated fluctuations. Figure 7a presents the shear stress–depth curves for different precompression strengths. The results indicate that as the precompression strength increases, the initial shear stress also increases; the shear stress decreases rapidly with depth at first, then the rate of decrease slows down and gradually stabilizes. Combined with the local magnified views, it can be seen that as the precompression strength increases from 0 MPa to 400 MPa, the magnitude of the stress drop decreases from 36 MPa to 26 MPa, and further decreases to 23 MPa when the precompression strength reaches 800 MPa.

Figure 7b shows the shear stress–depth curves for different precompression treatment times. The initial shear stress increases with increasing precompression treatment time, and the variation of shear stress with depth follows the same pattern as shown in Figure 7a. The inset shows that for precompression treatment times of 75 min and 150 min, the magnitude of the stress drop is 26 MPa and 45 MPa, respectively.

The stress drop observed in the load–depth curve essentially reflects the discrete evolution of localized plastic flow, specifically the dynamic process of shear band nucleation and explosive propagation [30]. Prior to the onset of shear instability, the system undergoes a quasi-static accumulation phase of elastic strain energy; once the critical threshold for nucleation is exceeded, the shear band undergoes unstable expansion, leading to an instantaneous and violent release of the accumulated energy. The amplitude of the stress drop, as characterized by the serration size, physically quantifies the local slip amount of a single shear event [31]: a larger amplitude indicates a more significant plastic displacement generated by a single shear band. This study reveals the dual regulatory effect of precompression parameters on the aforementioned dynamic behavior. As precompression intensity increases, the nucleation energy barrier of STZs within the material is effectively raised; this inhibitory effect on initial activation restricts the free evolution of slip displacement, manifesting macroscopically as a reduction in the amplitude of the stress jump. However, extending the precompression time induces a different structural response: although prolonged loading drives the matrix toward densification and increases its apparent hardness, this excessive homogenization actually weakens its ability to dissipate shear deformation. In the absence of effective geometric constraints or defect barriers, once a shear band initiates, it is highly prone to entering a catastrophic instability and propagation mode, leading to a surge in slip and ultimately manifesting as a rebound-like increase in the magnitude of stress drop.

As the fundamental physical carrier of localized plastic flow in MGs, the STZ serves as a key indicator of the strength of spatial synergy during atomic rearrangement at the microscopic level. The thermally activated nucleation of the STZ and its subsequent synergistic expansion together constitute the intrinsic dynamic mechanism underlying the nonlinear strain response of MGs. To gain a deeper understanding of how elastic precompression modulates the metastable topological structure and potential energy landscape of Ti-based MGs, this study introduces the CSM. By quantitatively inverting the STZ characteristic volume under different pretreatment conditions, the study aims to reveal the structural evolution induced by the prestress field at the atomic scale.

The Johnson–Samwer CSM [25] provides the core theoretical basis for the volumetric analysis ofSTZs within MGs. Based on the physical framework of this model, Pan et al. [32] developed a method for the quantitative calculation of STZ characteristic volumes in glassy systems using nanoindentation tests with varying loading rates. This model describes the key constitutive relationship governing plastic deformation behavior and establishes the relationship between the inelastic strain rate $\dot{\gamma }$ and the STZ activation energy barrier $W^{*}$ [32,33,34]:

(4) $$\dot{\gamma }={\dot{\gamma }}_{0}\exp(-\frac{W^{*}}{kT})$$

where $\dot{\gamma }$ is the inelastic strain rate, ${\dot{\gamma }}_{0}$ is a constant, k is the Boltzmann constant, and T is the absolute temperature. The expression for the STZ activation energy barrier $W^{*}$ is [32,33,34]:

(5) $$W^{*}=4\xi \Omega RG_{0}\gamma_{C}^{2}{\left(1-\frac{\tau }{\tau_{C}}\right)}^{3/2}$$

where ξ≈3 is a dimensionless constant, Ω is the STZ volume, R≈1/4 is a constant, $G_{0}$ is the Young’s modulus of the alloy at absolute zero, $\tau_{C}$ is the critical shear stress of the alloy at absolute zero, and $\gamma_{C}\approx 0.027$ is the average elastic limit. When the effect of normal stress is neglected, the activated volume $∆V^{*}$ is defined as [32,33,34]:

(6) $$\Delta V^{*}=-{\left(\frac{\partial W^{*}}{\partial \tau }\right)}_{P,T}$$

In nanoindentation experiments, $∆V^{*}$ can be used to express the activation volume of STZ as [32,33,34]:

(7) $$\Delta V^{*}=kT{\left(\frac{\partial \ln\dot{\gamma }}{\partial \tau }\right)}_{P,T}=\frac{kT}{\tau }{\left(\frac{\partial \ln\dot{\gamma }}{\partial \ln\tau }\right)}_{P,T}$$

In this equation, the physical meaning of $\frac{\partial ln\dot{\gamma }}{\partial \tau }$ is the reciprocal of the creep stress exponent m; therefore, the expression for the activation volume can be written as [32,33,34]:

(8) $$\Delta V^{*}=\frac{kT}{mt}$$

Combining Equations (5), (6) and (8) yields [32,33,34]:

(9) $$\Omega =\frac{kT}{G_{0}\gamma_{C}^{2}}\frac{1}{6\xi R}\frac{1}{m\frac{\tau }{\tau_{C}}{\left(1-\frac{\tau }{\tau_{C}}\right)}^{1/2}}$$

In the equation, the average elastic limit $\gamma_{C}$ is approximately 0.0267. In the Johnson–Samwer model [25], there is a general proportional relationship for the rheological stress at a given temperature: $\tau_{CT}/G_{T}=\gamma_{C0}-\gamma_{C1}{\left(T/T_{G}\right)}^{n}$, where $\tau_{CT}$ and $G_{T}$ are the critical shear strength and shear modulus at temperature T, respectively; $\gamma_{C0}=0.036$, $\gamma_{C1}=0.016$, and n=0.62. Therefore, the critical shear strength at 0K is $\tau_{C0}=0.036G_{0}$. Substituting the relationship between nanoindentation hardness and shear stress from Equation (1) into Equation (9) yields:

(10) $$\Omega =\frac{kT}{\frac{2R\xi G\gamma_{C}^{2}}{\sqrt{3}\tau_{0}}{\left(1-\frac{\tau }{\tau_{0}}\right)}^{1/2}mH}$$

The strain rate sensitivity index m is a critical parameter for calculating the STZ volume; it describes the degree to which the rheological stress of a material is sensitive to the strain rate during plastic deformation. The strain rate sensitivity index of MGs was characterized by measuring hardness values under different strain rate conditions using a step-rate test [32]:

(11) $$H=C_{1}{\left(\dot{\varepsilon }\right)}^{m}$$

In the equation, H represents the hardness at a constant strain; $C_{1}$ is a constant under a given stress condition; m is the strain rate sensitivity coefficient; and $\dot{\varepsilon }$ is the strain rate. In this experiment, the equivalent strain rate can be expressed as [32]:

(12) ε˙=h˙h=dhdth=P˙2P

In the equation, P represents the applied load.

We can rewrite Equation (11) in double logarithmic form:

(13) $$lnH=mln\dot{\varepsilon }+cont$$

**Figure 8:**
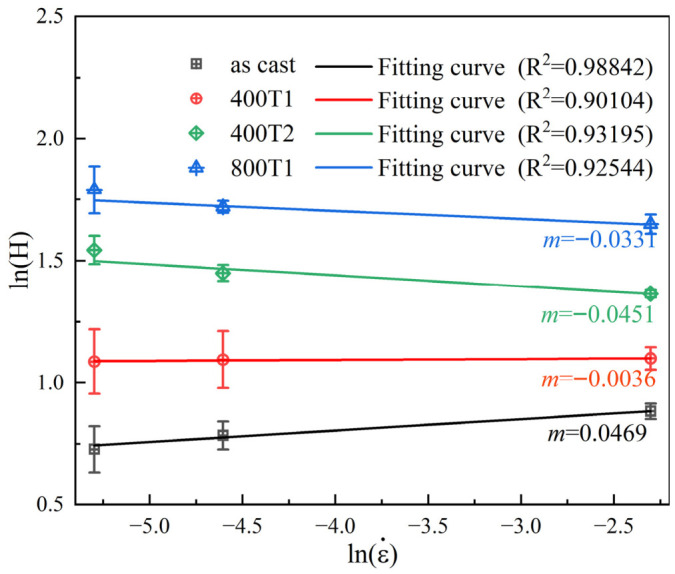
Double-logarithmic hardness–strain rate curves of different specimens.

Figure 8 shows the doubly logarithmic hardness–strain rate curves for different specimens; the strain rate sensitivity index m represents the slope of the linear fit curve. The resulting fitting curves agreed well with the experimental data, as confirmed by the goodness-of-fit (R^2^) > 0.98. The experimental results show that the as-cast sample exhibits positive sensitivity (m=0.0469), whereas the 400T1, 400T2, and 800T1 samples, after elastic precompression treatment, all exhibited a distinct “positive-to-negative reversal,” with their m values changing to −0.0036, −0.0451, and −0.0331, respectively. From a physical mechanism perspective, the positive or negative polarity of the m index directly reflects a fundamental shift in the plastic dynamics of the Ti-based amorphous matrix. A positive m value indicates that the material exhibits higher rheological resistance under high-rate loading; this rate-hardening effect effectively suppresses the explosive localization of shear bands, thereby inducing a more dispersed and uniform plastic deformation response in the micro-indentation field. Conversely, a negative m response induced by pretreatment signifies a transition of the plasticity mechanism from quasi-steady-state rheology to a shear band-dominated mechanism [6]. Under this mechanism, the material tends to release strain energy through the rapid nucleation and explosive propagation of shear bands.

The volumes of the shear transformation zone (STZ) derived for different loading rates are detailed in Table 1. As shown in the table, the STZ volume of the as-cast specimen is 1.524 nm^3^, corresponding to the peak value (0.0469) of the strain rate sensitivity |m|; for the 400T1 specimen, the STZ volume surged to 17.147 nm^3^, while |m| plummeted to 0.0036. This numerical interplay reveals the kinetic antagonism between STZ scale and rate response under the influence of the pre-compression stress field. Furthermore, the holding time also affects the STZ volume: when the holding duration was doubled from 75 min to 150 min, the STZ volume of the 400T2 specimen exhibited a significant “collapse” trend (decreasing to 1.963 nm^3^), while the sensitivity coefficient |m| rebounded to 0.0451.

Similar studies on inducing changes in the STZ volume of MGs through external factors have been reported. For example, Lv et al. [35] and Ma et al. [36] utilized Deep Cryogenic Cycle Treatment (DCT) to generate thermo-mechanical coupling in MGs, thereby triggering their non-equilibrium structural evolution and achieving regulation of the STZ volume. Lv et al. [35] reported that the STZ volume of a Ti-based MG first increased and then decreased with an increase in the number of deep cryogenic cycles, a trend similar to the results of this study. This demonstrates the effectiveness of stress-induced STZ volume regulation. Although the elemental compositions of the materials studied differ, the calculated STZ volumes are of the same order of magnitude, providing significant support for the validity and reliability of this study.

Based on the negative values of m for all pretreated samples (400T1, 400T2, and 800T1), and in accordance with the criteria established by Wang et al. [6], it can be concluded that their plastic flow is essentially governed by the explosive nucleation and propagation of shear bands. The discrete variation in |m| not only quantifies the response intensity of localized shear activity but also achieves a high degree of physical consistency with the characteristic volume evolution of STZ. The low coordinateness of the as-cast samples reflects their lower nucleation energy barrier, allowing atomic clusters at the microscopic scale to induce non-equilibrium deformation. For the 400T1 specimen, the mechanical rejuvenation effect triggered a global restructuring of the free volume, forcing the activation of STZ to overcome a larger-scale atomic coordination threshold, thereby constructing a more compact and homogeneous metastable structure. This rheological characteristic, by bringing m extremely close to zero, effectively suppresses localized instability in deformation, thereby inducing the formation of shear bands with the highest density during indentation cycles, that is, the most pronounced sawtooth events. As for the reduction in the STZ scale in 400T2 and 800T1, it profoundly reflects the competitive relationship between stress amplitude and the kinetic timescale in modulating the amorphous potential energy landscape.

## 4. Conclusions

In this work, elastic precompression was performed on Ti-based MGs at different loads and holding times. Nanoindentation results confirm that precompression induces a “decoupled” response characterized by increased hardness and decreased modulus. The STZ volume analysis revealed that the characteristic volume of STZ reached its peak, corresponding to an evolution of the sample’s serrated flow from a discrete step-like pattern to a high-frequency, low-amplitude wave-like pattern at a load of 400 MPa and a holding time of 75 min, which means that moderate prestressing can effectively raise the nucleation energy barrier of shear bands, thereby promoting the uniform nucleation and diffuse propagation of plastic carriers. Rate-sensitivity analysis further reveals that pre-treatment induces a sign reversal of the coefficient m, and the ∣m∣ value shows a significant negative correlation with the STZ size, reflecting the kinetic competition mechanism established by pre-compression between localized shear instability and coordinated atomic rearrangement.

## Figures and Tables

**Table 1:** Calculating the STZ volume using the rate of change method.

Samples	Pre-Compression Time (min)	m	Ω (nm^3^)
as cast	75	0.0469	1.524
400T1	75	−0.0036	17.147
400T2	150	−0.0451	1.963
800T1	75	−0.0331	3.069

Note: m—strain rate sensitivity coefficient; Ω—STZ volume.

## Data Availability

The original contributions presented in this study are included in the article. Further inquiries can be directed to the corresponding author.

## Abbreviations

The following abbreviations are used in this manuscript:

MGs	metallic glasses
CSM	Cooperative Shear Model
STZ	shear transition zone
MEMS	Micro-Electro-Mechanical Systems
